# The Situated Influence of Chronic Pain Perception on Chinese Older Adults’ Self-Management in Home Care

**DOI:** 10.3390/geriatrics3040064

**Published:** 2018-09-28

**Authors:** Fang Liu, Min Tong

**Affiliations:** School of Public Affairs, Xiamen University, No. 422 Siming South Road, Siming District, Xiamen 361005, China; mintong@xmu.edu.cn

**Keywords:** chronic pain perception, older adults, self-management, life modification

## Abstract

Background and objective: Worldwide, 26 million older adults die from chronic disease, and chronic pain is typically a part of the experience of chronic disease. This study explores the perception of chronic pain for home-dwelling Chinese older adults and its influence on (1) self-management ability and (2) management and reduction of chronic pain. Methods: Adopting a qualitative study design, we conducted in-depth interviews with 10 Chinese community-dwelling older adults who experience chronic pain. Half of our informants perceive chronic pain, whereas the other half, diagnosed with Alzheimer’s disease, do not report that they perceive chronic pain. Data were analyzed with inductive thematic analysis. Results: Chronic pain perception plays important roles in (1) defining the challenge of self-management, (2) connecting previous caretaking experience, (3) adjusting the identity of self-management, (4) acquiring support from important others and (5) re-planning self-management arrangements. Conclusion: Pain perception helps to motivate Chinese older adults to face health challenges and regain self-management capacity through adjustments in self-identity and care experience with the support of important others. Pain perception can consolidate the situation of independent living of older adults. It helps to motivate Chinese older adults to face health challenges and regain self-management capacity.

## 1. Introduction

Noncommunicable diseases, also known as chronic diseases, kill 41 million people each year, equivalent to 71% of all deaths globally. Of these, more than 26 million occur after the age of 70 [1]. To support each member state to better respond to chronic diseases, the World Health Organization has developed the Global Action Plan for the Prevention and Control of Noncommunicable Diseases 2013–2020. As part of the agenda, each country needs to develop a response to reduce the harm caused by chronic diseases through prevention and treatment [2]. As a member of the World Health Organization, China is experiencing an increase in longevity, which leads to more older adults with chronic diseases. More Chinese older adults are living with chronic diseases, and management of pain is an important emerging issue. In 2017, nearly 150 million older adults (≥60 years old) suffered from chronic diseases, accounting for 65% of the total elderly population [3]. In order to control the burden of chronic diseases effectively, the government developed the Medium- and Long-term Plan for Prevention and Treatment of Chronic Diseases in China (2017–2025). This plan is based on the living habits of Chinese older adults, that is 90% of older adults choose home-based care for chronic disease [4] and seem to prefer their own strategies developed in their daily home-based care, and their self-management strategies become a priority [5].

Some studies have shown that pain is not only a symptom or a pathogenic factor of chronic disease, it is also a disease entity [6,7]. Based on such studies, chronic pain can be understood as a factor and target of home-based self-management for older adults. In terms of factors, some studies have shown that pain perception has an obvious impact on disease management outcomes and treatment responses [8]. Most of these studies have used cluster analysis to identify subgroups of patients according to their pain perception characteristics [9]. These characteristics include adaptive differences, degree of injury, quality of life, and so on. Such characteristics can affect individual emotions, pain beliefs and treatment adherence of patients with chronic pain [10,11]. These studies do not clearly point out the relationship between pain perception and self-management. Recently, two studies clearly suggested that the classification of pain perception has a significant impact on patient self-management strategies. First, uncertainty of emotional distress and pain interpretation affect the interpretation of pain by patients with chronic pain [12]. Second, the perceived difference between chronic pain with neuropathic characteristics and chronic pain without such characteristics causes significant differences in the self-management experience [8].

The above studies indicate that different dimensions of pain perception affect the arrangement and effectiveness of patients’ self-management strategies. At the same time, subgroup comparisons in the pain perception dimension is a feasible research method. However, the existing research has the following shortcomings. First, most of the research is based on the premise that pain perception is regarded as a negative factor in a healthy life rather than a positive effect [13]. Second, due to the living environment and aging symptoms, older adults will emphasize their independence and adaptation to pain. Therefore, their pain perception is different from that of young adults [14]. Third, most of the research settings focus on nursing homes or hospitals rather than concrete home life situations [15]. Finally, it may be that patients with Alzheimer’s disease are unable to accurately report chronic pain [16], and their common behaviors associated with pain may be difficult to interpret [17]. Consequently, existing studies have paid little attention to pain perception in patients with Alzheimer’s disease [18]. In fact, the deficiency and even loss of pain perception in Alzheimer’s patients provides a meaningful classification. That is, older adults with chronic pain can perceive pain normally, and the pain perception of Alzheimer’s patients with chronic pain is obstructed or abnormal. Therefore, this study aimed to explore how pain perception affects home-based self-management by comparing two older adult subgroups with and without normal pain perception. We hope that the finding will change the service providers’ understanding of pain perception. In this way, service providers will provide self-management projects accurately for older adults with chronic pain.

## 2. Materials and Methods

### 2.1. Conceptual Definition

#### 2.1.1. Chronic Pain

The generally accepted definition of pain, as adopted in 1979 by the International Association for the Study of Pain, is an unpleasant sensory or emotional experience associated with actual or potential tissue damage or described in terms of such damage [6]. Pain is not only a simple physiological response, but also a personal subjective experience [19]. Everyone learns the application of the word through experience related to injury in early life [20]. Chronic pain can broadly be defined as pain that persists beyond normal tissue healing time, which is usually considered 3–6 months [21].

#### 2.1.2. Pain Perception

Pain perception consists of 3 aspects: sensation of pain, feeling of pain and sociocultural factors of pain. The sensation of pain is a physiological response that depends on the general level of organ pathology and noxious stimulation in the body [22]. The feeling of pain involves the processing of pain sensation by an individual’s mental model, which is a complex phenomenon caused by the interaction of motivational, emotional, cognitive and behavioral factors [19]. The sociocultural factors of pain relate to the definition and influence of cultural and social contexts on pain attitudes, meanings, norms and behavioral standards [23]. The biocultural model of pain perception proposed by Bates (1987) links sensation of pain and feeling of pain with social culture. Bates suggested that social comparison and social learning processes within situations go through family education and earlier pain experience to shape an individual’s attitude and behavior about pain. Attitude and behavior about pain cause the cognitive behavioral system to control the physical reactions to pain. When nociception goes beyond the critical value of cognitive behavioral control, an individual’s action expression system will show a response to pain. This action expression system may be different from the cognitive behavioral system, triggering a new round of cultural interpretation and experiential learning of pain [24]. In general, the physiological system of pain synchronously cooperates with the mental system and the social and cultural system in series, but this collaboration may be asynchronous in special conditions [23].

In fact, pain is a hypothetical concept that cannot be measured directly. Since some research has shown that the 3 systems of pain perception operate in tandem, pain perception can be expressed and explained by behaviors (verbal or nonverbal) that can be identified [19]. Therefore, these pain behaviors conform to social norms. In this sense, we can treat Alzheimer’s disease as a special condition in which the mental system is inoperable. The pain perception system of this patient group is out of order, so that they cannot express pain according to social norms or physiological reactions. Their pain perception is weakened by society or understood as an abnormal state. In recent years, research in the field of pain among Alzheimer’s patients has increasingly focused on understanding expressions indicating possible pain. The American Geriatrics Society suggests that the pain of Alzheimer’s patients can be expressed by changes in facial expression (e.g., frowning), vocalization and verbalization (e.g., groaning, mumbling) and body movements of agitation [18], even if these behavioral expressions do not conform to the social mainstream of pain expressions. Self-reporting is often considered as the gold standard in pain assessment [25]. However, most patients with Alzheimer’s disease cannot understand and answer questions raised in self-reporting, and proxy reporting is often a valuable option for this population [16].

#### 2.1.3. Older Adults’ Pain Self-Management

Self-management describes the skills and strategies that individuals use to take care of their health condition and minimize its impact on their lives [26]. Pain self-management for older adults is a multidimensional process that points to independent life. This process emphasize the decision-making and responsibility of older adults in painful daily life. Moreover, older adults should pay attention to the development of personal pain management technology and health knowledge [27]. The self-management of pain is not only an individual decision, but also involves the support of service providers and important others [28]. Especially when the cognitive function of older adults is impaired, it is more difficult for them to understand, remember and perform pain management [29].

### 2.2. Participants and Recruiting Procedure

In order to reduce the impact of environmental differences and regional culture on our research, we chose a densely populated community as our research unit. We worked with the community’s Geriatric Social Service Center, and the staff there recommended and contacted eligible respondents to participate in the study. The inclusion criteria were as follows: people aged ≥60 years old who have been diagnosed with chronic pain, participated in the self-management program provided by the community’s Senior Social Service Center for 2 years and lived at home at least for 2 years. In addition, the first subgroup of respondents had to satisfy the above criteria and score at least 17 points on the Mini-Mental State Examination to guarantee cognitive integrity (participants in this group were mostly illiterate or had primary school education). The second subgroup of respondents had to meet the same criteria and provide a diagnosis of Alzheimer’s disease and an assessment of cognitive disorder from a psychiatrist. Through the joint screening by the research team and the staff of the Geriatric Social Service Center, we obtained a group of 5 elderly dementia patients with chronic pain and 5 general elderly with chronic pain, as shown in Table 1. Written and verbal information about the study was provided. Respondents signed or put their fingerprint on the consent forms. As for the Alzheimer patients, their guardians had to sign and the patients themselves had to put their fingerprints in the presence of their guardians. In the course of the investigation, older adults usually report their self-care situation. According to the advice of nurses and social workers, the reports of the Alzheimer’s patients were provided by the major caregivers who registered at the Geriatric Service Center. Throughout the study, their rights were protected and identities kept private, and they were informed of their right to withdraw from the study at any time without detriment to their health care.

### 2.3. Data Collection

Because qualitative methods focus on people’s everyday lives, experiences and perceptions [14], we used semi-structured interviews to generate qualitative data. In order to reduce the impact of interviews on the daily life of participants, 3 interviews were arranged in their homes. The interviews centered on 3 aspects: physiological facts of pain, impacts of pain on daily household life and coping with pain. In order to have a more complete understanding of the participants’ pain story, we interviewed not only participants, but also their caregivers and important others, as well as nurses and social workers who provided services to them. Nurses and social workers have to participate in the self-care project of the Geriatric Service Center. They also have to serve the older adults who have been interviewed for at least 1 year.

### 2.4. Data Analysis

The data were analyzed with inductive thematic analysis. Thematic analysis is a flexible and useful method for understanding rich and detailed meaning within the situated data [30]. All interviews were transcribed verbatim by researchers who did not participate in the actual interviews and were read by the first author for transcript validation and contextual understanding. In the initial phase, the researchers coded line by line and extracted meaningful words about the research questions. Then, 2 authors reread the transcripts to elicit 58 initial nodes based on the discussion guide questions. Similar meaning nodes were then sorted into subthemes. Coding contents and naming were cross-examined and changed through consensus discussion between 2 authors. The final 24 nodes and 10 subthemes were merged into 5 themes. Examples of subsequent subthemes and nodes in acquiring support from important others are shown in Table 2. All authors critically reviewed and agreed on the themes. The final themes and subthemes are presented in Table 3. 

## 3. Results

### 3.1. Defining the Challenge of Self-Management

Older adults face various sorts of problems in their specific home living environment. In such an uncertain and complex context, older adults must accurately define the challenges of self-management so as to set relevant goals, arrangements and timing. Only in this way can they ensure that the self-management arrangements can be implemented smoothly, without being transferred or interrupted by accidents.

#### 3.1.1. Natural Aging

When pain occurs but cannot be clearly perceived, older adults are still in a home living state. Pain is a natural phenomenon of body aging rather than a health challenge that must be addressed. A self-management program will be carried out via an ordinary lifestyle, without a clear priority in life.

“She has no problem in life. At the rotation of eating and sleeping all day long, it’s natural for older adults to get sick,” Zhou’s husband said. 

“The caregivers report that it is quite normal that the aged always have some kind of pain, and it doesn’t deserve special attention,” Ting, a social worker, said. 

Pain and the challenges it brings do not receive enough attention. Under this circumstance, self-management arrangements fail in delaying physical deterioration and alleviating emotional distress. On the contrary, the demand for pain management in older adults has been neglected, seriously affecting quality of life. At the same time, older adults’ self-management experience and ability are naturally marginalized. 

#### 3.1.2. Health Warning

When an older adult perceives pain, he/she will jump out of his/her daily life state and find out the cause of the pain. Then, he/she will find out the health damage it indicates and reflect on his/her current life arrangements.

“When assessing self-management demands, we will ask older adults what the most urgent problem they need to deal with now. They will tell us something like relieving the pain, so we take this as the overriding goal,” Lin, a nurse, said. 

“When I felt a touch of rheumatic pain, I could not walk around by myself, nor go out to buy food. At that time, I have to find someone for help,” Yang, a patient, said. 

Perception of pain builds up a state of emergency in which the older adult pays attention to the challenge of pain and impaired life functions. Acting independently, going out to buy food and relieving pain are challenges that older adults are bound to face. However, resolving these challenges will also help to enhance their ability to cope with life problems and alleviate health risks caused by pain.

### 3.2. Connecting Previous Caretaking Experiences

Within Chinese culture, every change in the body can be explained by daily household life. Older adults often take unreasonable home life arrangements as the source of their pain. In other words, when perceiving pain, older adults will review their recent life arrangements and find aspects of their lifestyle that they consider unreasonable and unhealthy. Then, they will connect their previous home self-management experiences to modify the lifestyle and maintain their health.

#### 3.2.1. Other Experiences of Pain Management

When pain perception is impeded, older adults lose their connection with previous home self-management experiences. At the same time, explanations for pain based on life experiences also lose historical continuity. Older adults who have lost their care experience cannot cope with problems in their lives smoothly. They need care from caregivers. As a result, older adults’ self-management is passively connected with the life history and self-management experience of the caregiver.

“We also ask her to make bland, light meals, but she, with diabetes, always follow her own eating habits. My mother didn’t like many dishes she made,” Zeng’s daughter said.

“The nanny would not chat with him, and went to watch TV after simply fed him. After I came back, I found him speaking less and less, and his memory got even worse,” Chen’s wife said.

If the caregiver is a family member who lives with the older adult all year round, the life experiences of the two connect and overlap. Therefore, self-experience of the older adult is partially inherited by the caregiver, and his/her living habits and preferences may be satisfied. To a certain degree, older adults’ inherent self-management ability can be partially brought into play. If the caregiver is an employed non-family member, his or her experience may be far from that of the older adult. Life preferences and bad habits of the older adult are likely to be neglected. This neglect may give rise to further deterioration of the older adult’s physical and cognitive functions.

#### 3.2.2. Self-Experience of Pain Management

Although an older adult who can perceive pain well will accept other people’s advice, he/she prefers to connect the pain with his/her previous experiences in pain management. When pain occurs, the older adult can review and avoid repeating failed experiences in the past. Furthermore, he/she can cope with pain and the challenges it brings with his/her successful experience in self-management.

“If I have a headache, I will drink some tea, as it is said that tea can detoxify. I have had tea since childhood, so I am still in good health,” Shu said.

“I often feel a low back pain when it’s getting cold, and I will apply a homemade hot pack onto my waist and stop eating seafood,” Luo said.

As successful experiences continuously accumulate and failure experiences slowly decrease, the self-management ability of older adults is consistently enhanced. As a result, older adults can avoid the deterioration of physical and cognitive functions. At the same time, they can complete themselves via connections with past successful experiences, forming the wisdom of self-management.

### 3.3. Adjusting the Identity of Self-Management

A vital attribute of self-management is the actively participating subject, and many older adults with chronic pain play a part in self-management arrangements [20]. Active participation in arrangements means that older adults can gain control over their bodies and lives. Older adults transform from passive objects of pain treatment to subjects with power, and their individual experiences and abilities thus derive value and significance [31]. Since active participation can lead to value and significance, older adults’ willingness to learn is stimulated. Because older adults actively learn pain management skills, their health care knowledge is thereby developed. Meanwhile, physical injury and psychological distress can be controlled within acceptable limits [25].

#### 3.3.1. Objects of Pain Management

Older adults with impaired pain perception can express their pain via facial expressions (such as frowning), seemingly meaningless verbal expressions, aggressive words or intense body motions. Such expression may exceed caregivers’ or service providers’ general understanding of pain perception. More so, such expression may be passively connected with non-painful abnormalities in mainstream social discourse. Then, their requests for pain management will be ignored or misunderstood. 

“When he had a headache, he would tell us that he felt dizzy, but I didn’t know where he had the problem or other symptoms. So I asked him to take medicine of coronary heart disease first and others if it doesn’t work. I gave him tranquilizers if he kicked up a row, and then he would go to bed,” Xie’s wife said. 

“When older adults feel hurt, they will communicate with you via body language. With careful observation and summary, you can understand what they mean. However, many caregivers will neither carefully observe nor record the pain expression of Alzheimer’s patients. They always arrange older adults’ lives in terms of their own thoughts,” Ting, a social worker, and Lin, a nurse, said.

When pain requests are considered invalid, older adults lose the power to manage their own pain. As a guardian, caregivers inherit the power of older adults. Caregivers will judge whether the older adults have pain and what kind of treatment is needed based on their own understanding. Older adults, just like puppets in the hands of caregivers, will feel powerless. 

#### 3.3.2. Subjects of Pain Management

Older adults with clear pain perception can complete connections between pain input, early social learning and pain experience before expressing their pain. They know what they are facing and what kind of help they need. Meanwhile, they can use language or text to express pain and ask for help. Caregivers and service providers can understand requests of older adults through communication rather than subjective conjecture. In this way, the possibility that pain requests may be ignored and misunderstood is greatly reduced. 

“My right leg hurts when it rains, so I explained this to the family doctor. He suggested that I take anti-inflammatory painkillers, which, however, would make my stomach hurt, so I bought notoginseng powder. After having taken for a period of time, my right leg didn’t hurt much in rainy days,” Huang said.

Through clear pain expressions and requests, older adults’ pain management experience can be given some attention and concern. Thus, they can have the subjective discourse power of handling their own pain problems. Especially when older adults have basic self-management ability, they will acquire stronger decision-making power for self-management arrangements. Consequently, older adults become experts in solving their own pain.

### 3.4. Acquiring Support from Important others

Establishing mutual trust and mutual support between older adults and caregivers and service providers is a key factor in pain management [32]. For older adults, the most important support stems from important others, such as caregivers, with life interactions and shared life memories. Ideally, important others can listen to the needs of older adults and pay attention to their habits. They can also respect older adults’ preferences and provide suggestions for change related to their individual lives. With respect and support of important others, older adults will more actively adjust their emotional distress and meet the challenge of pain. Furthermore, older adults will also believe in their own care service provider taking ability. 

#### 3.4.1. High-Frequency and Low-Association Support

In order to reduce the possibility of falls and prevent accidents, older adults whose pain perception is impaired should live with at least two caregivers, as shown in Table 1. Caregivers will make a plan to ensure that the older adults are in custody 24 h a day. In the meantime, other important family members may visit frequently to care for the health condition of the older adult. However, on the one hand, older adults with impaired pain perception cannot clearly express their pain challenges and emotional distress to important others; on the other hand, important others may take no account of or misinterpret the signals sent by the older adult. Although older adults have received more attention, this concern does not match their real needs. In low-association care, older adults’ physical pain warning fails to receive timely attention and treatment, and lesions will potentially occur.

“When I applied ointment to decubitus on his feet, it might hurt, and he would start to swear. He would even kick me, just like an insane. Now he has to recuperate in bed, so I feel less saddled and I can also go out,” Xie’s wife said.

“I have to stay at her house and watch her in case she falls or does something else dangerous. I can only go out when her husband comes home. She didn’t talk all day long, so I made the meal in terms of my own idea,” Zhou’s nanny said. 

Twenty four-hour monitoring also means that older adults are likely to lose the opportunity for social communication and support training. Consequently, their language function is weaker, and they will be more indifferent to the surrounding environment. What is worse, older adults adopt abnormal pain expressions, including abnormal behaviors and spatiotemporal disorders. They will produce harmful effects on the lives of others and the public space. Without the support of an explicit medical diagnosis, some caregivers treat such older adults as patients with mental illness. In this case, the older adults are considered by the caregivers as a burden instead of important family members. They are dedicated to tackling the abnormal state of older adults to diminish social impacts, rather than assisting older adults in expressing and managing their pain.

#### 3.4.2. Low-Frequency and High-Association Support

Older adults with clear pain perception will express their physical pain and emotional distress in a way that can be understood and accepted by important others. Therefore, important others can obtain understanding of and support for their physical and emotional status. However, older adults with better pain perception seem to be more inclined to live alone or with a spouse, as shown in Table 1. Children only visit older adults during holidays. Even if there are important people living together, the older adults do not need intensive and long-term care.

“The kids are working in other cities, and they only have time to come back to see us during holidays, two or three times a year. We also hope that we can take care of ourselves and not interfere with our children’s work,” Yang said.

“My daughter knew that my arthritis hurts badly, so she asked me to bathe my feet and massage the soles before sleep. She even bought a foot bath tub for me,” Yu said.

On the one hand, since older adults can clearly express their pain, important others will provide support according to their needs. On the other hand, older adults will also ask important others to support them according to their situation. Important others will provide older adults with resources accurately to resolve self-management challenges, which include health care products, healthy life knowledge, emotional support and spiritual comfort. At the same time, older adults acquire affection and dignity in the course of communicating with important others. That will be an important driving force for older adults to improve their self-management arrangements continuously and have concern for their own life value.

### 3.5. Re-Planning Self-Management Arrangements

As mentioned above, older adults see recent unreasonable home life arrangements as a source of pain. When they find a pain explanation from life, they will re-plan their self-management arrangements. When older adults arrange their lives, they prefer low-cost self-management with little change that fits their own living habits [5]; for instance, improving dietary structure and changing sleep time. When new self-management arrangements begin to take effect or bring better experience to older adults, they will gain the motivation to continue to implement them. Moreover, older adults will develop a new healthy lifestyle, thereby reducing their risk of disease or disability.

#### 3.5.1. Vicious Circle

Older adults with impaired pain perception cannot identify their challenges. In the meantime, they have no power and support to rearrange their lives conducive to body recovery based on the challenges of physical pain and self-management experience. 

“For us, the biggest difficulty in providing services for caregivers of Alzheimer’s patients is unreasonable drug use and dietary arrangements. We would like to advise the caretakers to rearrange them, and they agreed in our presence. However, after returning to real life, they still abuse drugs to control some excesses of older adults, or make some meals with heavy oil and salt,” Ting, a social worker, and Lin, a nurse, said. 

“In a few days, he was particularly out of temper, and sometimes rolled around on the bed, but I didn’t pay much attention. Ten days later, he suddenly had a fever and foamed at the mouth. I could only hasten to send him to the hospital,” Xie’s wife said.

Caregivers cannot discover defects in the living arrangements until the older adults’ physical deterioration reaches obvious harm. Existing care arrangements based on caregivers’ own experiences may replicate risk factors and exacerbate the physical and cognitive state of older adults. In this case, minor changes brought by home care arrangements may no longer be effective. Older adults and their families have to face the plight resulting from high medical costs and scarce medical resources. 

#### 3.5.2. Benign Change

Older adults with clear pain perception can detect physical injuries caused by poor living arrangements through pain pre-warning. With the support of important others, they can re-plan home self-management arrangements in terms of previous care-taking experience or suggestions from others.

“I often have insomnia. Later, the social worker advised me to take vitamin B to promote sleep. I bought a bottle, which costed only two yuan. Then I can sleep well at night,” Huang said.

“Previously, I was used to doing exercises with an empty belly in the morning. I fell once in running because of low blood pressure. Later, my husband would prepare me a cake or cereal to supplement my physical strength before I go out to run early every morning,” Shu said. 

Re-planning self-management arrangements are a kind of lifestyle modification, low-cost, easy-to-operate and efficacious. If lifestyle modification can effectively relieve pain, older adults will reduce drug dependence and drug abuse. In the meantime, pain relief can effectively prevent further deterioration of physical damage and reduce the hospital admission rate. Besides, older adults can continuously learn and integrate their self-management skills and healthy life knowledge in the process of re-planning. 

## 4. Discussion

The respondents told about their experiences dealing with chronic pain in home-based situations. The results revealed that older people with clear pain perception were able to identify the health risks indicated by the pain. Therefore, they could set goals for self-management plans. To achieve the goals, these older people rearranged their life by utilizing their own experience, consolidating the support of important others and playing the subjectivity role. On the contrary, because of impaired pain perception, Alzheimer’s patients’ pain reactions were considered as natural aging phenomena. Therefore, their self-management goals were extensive and vague. At the same time, Alzheimer’s patients became the objects of care by others. Although they could gain high-frequency support from relatives, this support was not very relevant to their needs. Self-management without goals and subjectivity might neglect current health risks or even exacerbate the health problems of older adults, which will eventually bring about critical disease or disability.

As a matter of fact, a self-management plan is a kind of arrangement of daily life for older adults. Clear pain perception can reinforce older people’s situation of living independently in five aspects: defining challenges, connecting experiences, adjusting identity, acquiring support and re-planning arrangements. The independent living situation after reinforcement offers advantages for the self-management plan. Meanwhile, the self-management plan also conforms to the wishes of older people to live independently and improves their ability to deal with life issues. In Chinese culture, one Confucian value is self-sacrifice [33]. Influenced by their current sense of worth, older adults would rather live independently than become a burden to others [34]. Older adults whose pain perception is hindered experience a sense of brokenness and dependence on care. They are apt to live together instead of living independently, which challenges the intrinsic individual service orientation of a self-management plan. Research also found that it was easier for older adults whose pain perception was hindered to gain 24-h surveillance by important family members. Therefore, family is essential to help older people.

In most existing research, pain perception has been a noxious element that affects the physical and mental health of older adults. Furthermore, pain perception is the natural enemy of self-management plans. However, this study found that pain perception had a promoting effect on self-management plans. We will further explore the reason why clear pain perception can sustain an independent living situation for older adults in China and supply advantages for self-management plans.

Existing research on risk enablement of Alzheimer’s disease sufferers interprets the mechanism of pain perception to some certain degree [17]. If we regard pain as a warning about one’s mental and physical health, then we can also consider self-management of chronic pain as a type of risk management. According to the proposition of risk enablement, risks and opportunities coexist. If Alzheimer’s disease sufferers proactively participate in risk communication and management, they are able to manage risks, act on their own and realize self-growth [35]. Therefore, Alzheimer’s disease sufferers would be free of control by caregivers and experts [36]. As a result, risk enablement promotes self-empowerment, which is the core viewpoint of this concept. 

Serving as an early warning of risk, pain perception can preserve older adults’ ability to live independently through resource integration and risk prevention. Maintaining independent living is actually a self-empowerment process and advances the implementation of self-management plans. However, the logic behind the concept of self-empowerment was not further explored in the risk-enhancement studies. Therefore, this study tries to search for the meaning of self-empowerment from the standpoint of self. Modernist writers hold that the self is an independent substantial being with an unchanged nature, which has thoughts regardless of the existence of the body [36,37,38]. Therefore, an individual is a conscious, independent and rational person with the body and thoughts separate. Postmodernist authors, on the other hand, believe that “there is nothing outside of the text.” They thoroughly deny the independence of self or the subject as an entity, and the self thus transforms into a non-subjective and fragmented one [39,40].

Pain perception helps older adults who suffer from chronic pain find themselves. This “self” means neither the intrinsic self with a separation of body and mind in modern discourse, nor the rootless self-detached from situations in postmodern discourse. Most of the existing pain studies found a biopsychosocial model based on the modern concept of self [41]. In this model, older adults in pain lack scientific reasoning and need experts to take care of their bodies [42]. Psychiatrists should be answerable to psychological pain, and surgeons should be answerable to bodily pain. This means that the pain experience of older adults is marginalized, and they no longer need the capacity to manage themselves [42]. The self in post-modern society broke away from the concrete life situation and just existed in the bitter narration of all kinds of media reports [43]. Older adults are busy accepting the explanations relevant to pain. However, they only discover that they lack concrete pain management plans in their everyday lives [44]. Older adults are busy ascribing pain to others’ wrong proposals and the deficiency of medical institutions. On the other hand, they always absolve themselves from responsibility for pain in everyday life [45].

Compared with the modern self and postmodern self in Western philosophy, self in Chinese traditional culture means an entirety that includes both body and mind [46]. It is a present state that can echo with time and space, namely the concrete reflection of situationalization in Chinese culture [47]. Generally, older adults in China accept this kind of self, and they always arrange their daily life on the basis of this concept [48]. Pain perception makes older patients with chronic pain gradually conscious of self-imbalance. Then, they will choose to live independently away from the interference of others. They will observe their own bodies and minds, as well as the outside world. They will explore the situation that they ignored before [49]. They do not consider strict biochemical indicators as the proof of pain [47]. They are fully confident in their physical experience and subjective feelings in the present situation and manage their pain on the basis of those experiences and feelings. They improve their abilities in the process of improving their own lives [50]. Especially when they live alone, their physical and mental experience becomes the norm for estimating risks. Older adults themselves become the experts of their physical pain and are responsible for their own pain.

Therefore, when older adults in China perceive pain in everyday life, they will open up a situation of independent living. Then, they detect and reflect the whole self-state in the situation. During the process of reflecting and improving the self, they can gain more experience and are more conscientious. This is the logic of self-empowerment. Furthermore, this is an essential qualification if one wants to realize self-management plans. Because of the shortage of medical resources and the large population of older adults, China’s pension system is confronted with an imbalance of supply and demand of medical resources [51]. To solve this problem, the Chinese government advocated the use of Chinese medicine knowledge for self-management at home, which is based on the concept of the whole self [52]. The purpose of advocacy is for older adults to accept using this knowledge in their daily lives and even decrease the possibility of disease or disability caused by pain [53]. At the same time, the Chinese government wants to reduce the burden of family care and social medical care and to relieve the shortage of medical resources [54].

This study had several limitations. First, participants were exclusively urban dwellers. The results may therefore not apply to other populations of older persons with chronic pain. Second, inductive thematic analysis is affected by the researcher’s own understanding of pain. We hope to reduce the influence of the researcher’s inherent subjectivity by having respondents examine the report of the research results. Third, in the absence of self-reports, the interpretation of pain by a significant other is questionable. Proxy reports can only be legitimated if significant others’ perceptions are comparable to the Alzheimer patients’ own perceptions of pain. In order to get the proxy report close to the self-report, we tried our best to interview more important others in addition to the agents as much as possible to understand the whole pain story of participants. Fourth, in this study, we found that the obstruction of pain perception will shape the family life situation, in which the self-management plan will reconsider the previous individual service orientation. This paper focuses on the positive effects of pain perception without further discussing the above findings. In future studies, we will delve into the management of pain in family life situations for Alzheimer’s patients.

## 5. Conclusions

Pain perception consolidates the situation of independent living of older adults in five aspects: defining challenges, connecting experiences, adjusting identity, acquiring support and re-planning arrangements. Then, it can provide advantages for the home-based self-management of older adults in China. This finding differs from previous studies related to pain perception, which considered pain perception as an adverse factor of disability and morbidity. The promoting effect of pain perception on independent living is rooted in examination of the whole self in Chinese traditional culture. Examination of the whole self can increase older adults’ power by enriching their experience and raising their responsibility.

To enable service providers to design and deliver more effective self-management projects, we propose the following suggestions to service providers. First of all, service providers must pay attention to the pain expressions of older adults, even if the expression lacks vocabulary and is repetitious. The challenges faced by older adults in self-management should be determined based on their pain perception rather than expert advice. Second, it is necessary for service providers to respect the subjective identity and care experience of older adults in their self-management at home. They are experts on their own bodies rather than passive, ignorant, lifeless puppets. Older adults can be responsible for their own bodies. Third, service providers must see the self-growth of older adults as the ultimate service goal, motivating them to continuously create and update self-management knowledge. Finally, service providers must fully mobilize important others to provide material and emotional support for the self-management of older adults. This support should be based on the understanding of older adults’ pain expression and respect for their self-determination, rather than on the important others’ own pain interpretation system. Thus, self-management projects must aim to maintain an independent living situation for older adults. Service providers must be alert to the weakening of older adults’ abilities by social stereotypes and professionalism.

All subjects gave their informed consent for inclusion before they participated in the study. The study was conducted in accordance with the Declaration of Helsinki.

## Figures and Tables

**Table 1:** Characteristics of study participants (*n* = 10).

Participant Pseudonym	Sex	Age (Years)	Illness Condition		Important Others	Living Situation	Social Worker	Nurse	Report
			Alzheimer’s (Yes/No)	Chronic Pain					
Wu	Female	93	Yes	Femur fracture, bedsores	Son	Family life	Ting	Lin	Proxy report by son
Xie	Male	81	Yes	Low back pain, bedsores	Wife	Family life	Ting	Lin	Proxy report by wife
Zeng	Female	88	Yes	Shoulder periarthritis, arthritis	Daughter, nanny	Family life	Ting	Lin	Proxy report by daughter
Chen	Male	71	Yes	Tibia fracture, back pain	Wife, nanny	Family life	Ting	Zhuang	Proxy report by wife
Zhou	Female	79	Yes	Rheumatoid arthritis	Husband, nanny	Family life	Ting	Zhuang	Proxy report by husband
Huang	Female	93	No	Right leg fracture, rheumatoid arthritis	Nanny	Non-family care life	Ting	Lin	Self-report
Shu	Female	74	No	Shoulder periarthritis	Husband	Family life	Ting	Lin	Self-report
Yang	Female	95	No	Rheumatoid arthritis	Daughter	Living alone	Ting	Lin	Self-report
Luo	Female	83	No	Low back pain		Living alone	Ting	Lin	Self-report
Yu	Male	83	No	Arthritis		Living alone	Ting	Zhuang	Self-report

**Table 2:** Subthemes and nodes of acquiring support from important others.

Subthemes	Final Nodes	Initial Nodes
High-frequency and low-association support	High-frequency support	Seniors live under 24-h monitoring by caregivers
		Visits from other important family members once or twice a week
		Live and eat together every day
	Low-association support	Needs of older adults are misrepresented
		Older adults are considered mentally abnormal
		Older adults are seen as a burden
		Caregivers arrange their lives according to their preferences
Low-frequency and high-association support	Low-frequency support	Older adults live alone
		Older adults travel alone
		Visits from family members during holidays
	High-association support	Older adults put forward their own needs
		Older adults reject help they do not need
		Important others provide assistance according to the needs of older adults
		Older adults provide feedback to important others on their effectiveness

**Table 3:** Effects of pain perception on self-management.

Themes	Subthemes
Defining the challenge of self-management	Natural aging
	Health warning
Connecting previous caretaking experiences	Other experiences of pain management
	Self-experience of pain management
Adjusting the identity of self-management	Object of pain management
	Subject of pain management
Acquiring support from important others	High-frequency and low-association support
	Low-frequency and high-association support
Re-planning self-management arrangements	Vicious circle
	Benign change
